# Subarachnoid haemorrhage or traumatic lumbar puncture. Differentiation by cerebrospinal fluid parameters in a multivariable approach

**DOI:** 10.1038/s41598-023-49693-y

**Published:** 2023-12-15

**Authors:** Anne Zinganell, Klaus Berek, Gabriel Bsteh, Franziska Di Pauli, Verena Rass, Raimund Helbok, Janette Walde, Florian Deisenhammer, Harald Hegen

**Affiliations:** 1 Department of Neurology, Medical University of Innsbruck, Anichstrasse 35, 6020 Innsbruck, Austria; 2 Department of Neurology, Medical University of Vienna, Vienna, Austria; 3 Department of Neurology, Johannes Kepler University, Linz, Austria; 4 Department of Statistics, Faculty of Economics and Statistics, University of Innsbruck, Innsbruck, Austria

**Keywords:** Stroke, Blood-brain barrier

## Abstract

Lumbar puncture (LP) is recommended in patients with thunderclap headache and negative computed tomography to rule out spontaneous subarachnoid haemorrhage (SAH). Blood contamination of cerebrospinal fluid (CSF) due to traumatic LP poses a diagnostic dilemma. Therefore, routine CSF parameters were investigated to distinguish between SAH and a traumatic LP. CSF red blood cell (RBC), white blood cell (WBC) count, total protein, CSF colour and supernatant were used for group comparisons of patients with SAH and ‘symptomatic controls’. Due to variable time intervals between bleeding onset and LP in SAH patients in contrast to patients with traumatic LP, where blood contamination of CSF occurs at the time of LP, CSF variables were adjusted for decay in time to allow comparability. Logistic regression analysis identified bloody CSF [odds ratio (OR) 32.6], xanthochromic supernatant [OR 15.5] and WBC_adjusted_ [OR 4.5 (per increase of 100/µl)] as predictors of SAH, while age, sex and CSF total protein_adjusted_ were no predictors. Optimal cut-point of RBC_adjusted_ (determined at day 1 after bleeding) was > 3667/µl to identify SAH patients with a 97% sensitivity and 94% specificity. Combination of low RBC and clear CSF supernatant was found in none of SAH patients. Combined CSF RBC count and CSF supernatant reliably distinguished traumatic LP from SAH.

## Introduction

Spontaneous subarachnoid haemorrhage (SAH) is a severe life-threating neurological disease which accounts for approximately 5–10% of all strokes and bears the risk of significant morbidity and mortality^1,2^. Cerebral computed tomography (CT) scan is the first investigation if SAH is suspected. Its diagnostic sensitivity is high in the first hours after the bleeding, but sharply decreases thereafter^3,4^. As patients frequently present hours or even days after symptom onset, lumbar puncture (LP) should be performed in case of normal CT scans to detect the low but clinically significant percentage of CT negative SAH patients^5^. Under physiological conditions, cerebrospinal fluid (CSF) does not contain red blood cells (RBC) and, thus, can rule out SAH. However, RBC are artificially introduced into the CSF in up to one third of patients as a result of needle trauma mostly due to puncturing spinal venous plexus^6^. This hampers the differentiation between patients with SAH and traumatic LP.

Prior studies have investigated various CSF biomarkers to identify SAH patients, e.g., RBC count, xanthochromia, oxyhaemoglobin, methaemoglobin, ferritin or siderophages, but reported only moderate diagnostic value due to various reasons^7^. Furthermore, some of these biomarkers are not in wide clinical use. While different temporal dynamics of CSF biomarkers in SAH patients have been acknowledged and certain time frames defined for their detection, e.g., bilirubin > 12 h after the bleeding^7^, none of these studies considered that the magnitude of CSF changes might also depend on disease duration.

The objective of this study was to investigate the diagnostic value of widely available CSF parameters to discriminate patients with SAH from patients with diseases other than SAH but with traumatic LP applying a multivariable approach and considering disease duration as a covariate in particular. In addition, a literature review was conducted to provide an overview of current knowledge on the ability of CSF parameters to differentiate between SAH and traumatic LP.

## Methods

### Patients and samples

We have stored the results of CSF and serum sample analyses performed for routine diagnostic purposes in the Innsbruck CSF Database from patients with mainly neurological diseases. We screened for samples applying the following criteria: CSF collection by LP and sample processing within 24 h after withdrawal (done on Nov 6th, 2022). Medical records of the remaining patients were studied and patients classified into a spontaneous SAH group and control group. The authors had access to information that could identify individual participants during or after data collection.

**Figure 1:**
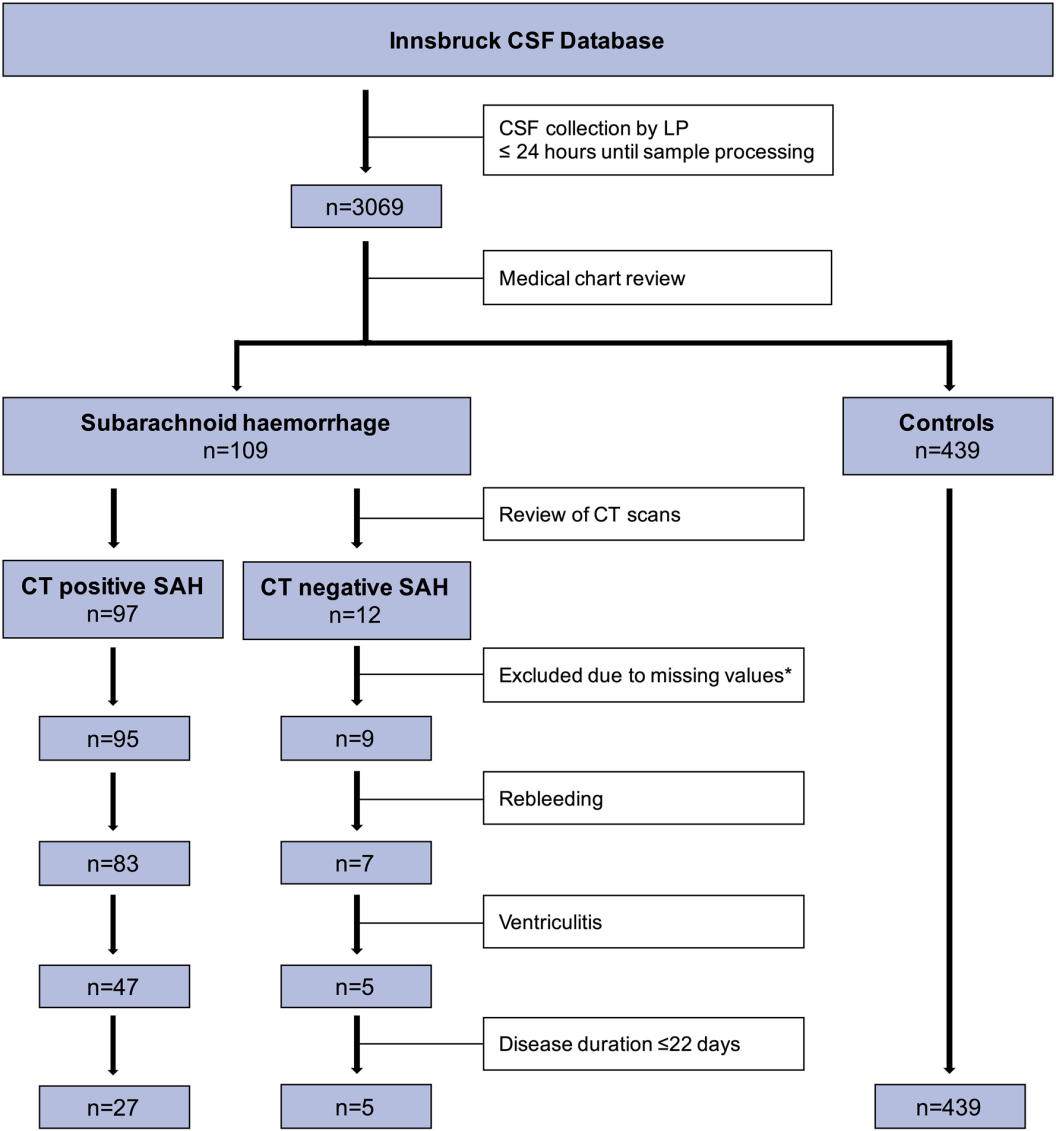
Sample flow chart by various inclusion criteria. * of CSF total protein or CSF supernatant. *CSF* cerebrospinal fluid, *CT* computed tomography, *LP* lumbar puncture, *n* number, *SAH* subarachnoid haemorrhage.

Patients with spontaneous SAH were subdivided into patients with evidence of subarachnoid blood on the CT scan (termed ‘SAH’) and patients without evidence of intrathecal blood on the CT scan (termed ‘CT negative SAH’). Within the SAH and CT negative SAH group, patients with re-bleeding or ventriculitis before LP were excluded to ensure that adjustment for disease duration (see below) is not biased by these complications, which might lead to changes in RBC, WBC count and CSF total protein (Fig. 1).

Patients fulfilling the definition of ‘symptomatic controls’ according to a recent consensus^8^ or patients without any neurological disease were used as control group. These patients should show normal routine CSF findings (i.e. normal WBC count and CSF total protein). Diagnoses of the control group are given in Table S1.

### Definition of CT negative SAH

Patients classified as ‘CT negative SAH’ had typical clinical presentation (thunderclap headache), normal CT scan, but further clinical/ paraclinical findings, which led to the diagnosis of ‘CT negative SAH’ by the treating physicians, e.g., MRI with evidence of bleeding, signs of erythrophagocytosis in the CSF (detection of erythrophages, siderophages, hematoidin and/ or hemosiderin).

### CSF analysis

CSF WBC and CSF RBC were counted within a Fuchs-Rosenthal chamber, which has a volume of 3.2 μL^9^. Division by 3.2 allowed reporting of cell counts per μL according to International System of units (SI). CSF total protein concentration was measured by spectrophotometry after incubation of centrifuged CSF with 3% trichloroacetic acid^10^. CSF colour (clear, bloody) and CSF supernatant (clear vs. xanthochromic) were classified by visual inspection.

### Statistical analysis

Data were displayed as mean ± standard deviation (SD) or as median, interquartile range (IQR), 5th-95th percentile, as appropriate.

As CSF of patients with SAH is usually taken hours or days after the bleeding, in contrast to patients with traumatic LP, where blood contamination of CSF occurs at the time of LP, the variables RBC count, WBC count and CSF total protein were corrected for their decay in time to make them comparable between patients. They were adjusted according to the decay law previously published^11^.

$${y}_{adjusted}={y}_{{T}_{i}}\cdot {e}^{-\widehat{\lambda }\cdot \left({T}_{i}-1\right)}$$

where $y$ denotes RBC, WBC or CSF total protein respectively, ${T}_{i}$ is the disease duration (days since symptom onset) for patient $i$, and $\widehat{\lambda }$ is the estimated constant time decay parameter. For our purpose the three variables (RBC, WBC or CSF total protein, respectively) were adjusted to ${T}_{i}=1$, and we used only patients who had LP less or equal than 22 days after disease onset (Fig. 1), as the decay parameter was estimated for this time period in the previous study^11^. The $\widehat{\lambda }$ for RBC, WBC or CSF total protein were -0.281, -0.217 and -0.063^11^. E.g., a patient with a RBC count of 2000/μl determined in CSF collected three days after SAH onset has an RBC_adjusted_ of 3508/μl, calculated as

$${RBC}_{adjusted}=2000\cdot {e}^{0.281\cdot \left(3-1\right)}$$

With a binary logistic regression model the probability for both SAH & CT negative SAH was modelled using RBC_adjusted_, WBC_adjusted_, CSF total protein_adjusted_, CSF supernatant, CSF colour, sex and age as independent variables. As sample size was small a likelihood ratio test was used to test for statistical significance of each variable.

We calculated the optimal cut-off point in the logistic regression of the probability of both SAH & CT negative SAH dependent on RBC_adjusted_, using the sum of specificity and sensitivity as optimization criterion.

The significance level was 5%. All computations were done with R Core Team (2017) and the package cutpoint^12,13^.

### Ethics

The conduct of the study was approved by the Ethics Committee of the Medical University of Innsbruck (approval number 1269/2022). Informed consent was not needed because this was a retrospective analysis of existing data that were obtained in routine diagnostic procedures.

### Literature search

A literature search in PubMed using the search terms “cerebrospinal fluid” AND “subarachnoid haemorrhage” AND “traumatic tap” or “red blood cell count” or “white blood cell count” or “total protein” or “opening pressure” or “three tube test” or “xanthochromia” or “colour” or “supernatant” or “ferritin” or “oxyhaemoglobin” or “bilirubin” or “methaemoglobin” limited to August 1st, 2022 offered 18, 39, 35, 80, 32, 7, 92, 25, 17, 24, 70, 119, and 17 references. Abstracts that did not primarily deal with the use of CSF to differentiate between SAH and traumatic LP were excluded. In addition, articles identified in reference lists of individual papers were selected if considered appropriate. Only original articles written in English were considered.

## Results

### Routine CSF parameters reliably discriminate SAH from traumatic LP

A total of 471 samples comprising 27 patients with SAH, 5 patients with CT negative SAH and 439 controls were included in the study. For inclusion criteria see Fig. 1. Demographic and clinical characteristics of the three groups are detailed in Table 1, the main CSF findings are shown in Table S2, a detailed characterisation of the CT negative SAH patients is provided in Table S3.

**Table 1:** Demographic and clinical characteristics.

	SAH	CT negative SAH^c^	Controls
N	27	5	439
Sex (female), n (%)	13 (48)	4 (80)	181 (41)
Age (years), mean ± SD	62 ± 12	42 ± 11	44 ± 16
EVD^a^, n (%)	9 (33.3)	0 (0)	NA
Disease duration^b^ (days), median (IQR)	9 (4.5–16)	6 (1–8)	NA
Aneurysm detection, n (%)	17 (63)	2 (40)	NA
Intervention, n (%)	17 (100)	2 (100)	NA
Clipping, n (%)	8 (47)	1 (50)	NA
Coiling, n (%)	9 (53)	1 (50)	NA
Time interval between LP and sample processing (hours), median (IQR)	0.8 (0.3–2.0)	0.8 (0.6–0.9)	0.6 (0.4–1.5)

^a^Number of patients who required insertion of EVD during the disease course (before CSF collection by LP).

^b^Disease duration was defined as the time interval between symptom onset and LP.

^c^Diagnosis of CT negative SAH was based on typical presentation (thunderclap headache, n = 5), normal CT scan, but further clinical/ paraclinical findings which lead to the diagnosis of ‘CT negative SAH’ by the treating physicians (Table S3).

*CT* computed tomography, *EVD* external ventricular drainage, *IQR* interquartile range, *LP* lumbar puncture, *n* number, *NA* not applicable, *SAH* subarachnoid haemorrhage, *SC* symptomatic controls, *SD* standard deviation.

The median disease duration, i.e. the time interval from symptom onset to LP, in patients with SAH was 9 days and in patients with CT negative SAH 6 days. We adjusted RBC, WBC count and CSF total protein for the individual disease duration, i.e. the time interval from symptom onset until LP. For comparison of the measured and adjusted values, please refer to Table S2.

**Figure 2:**
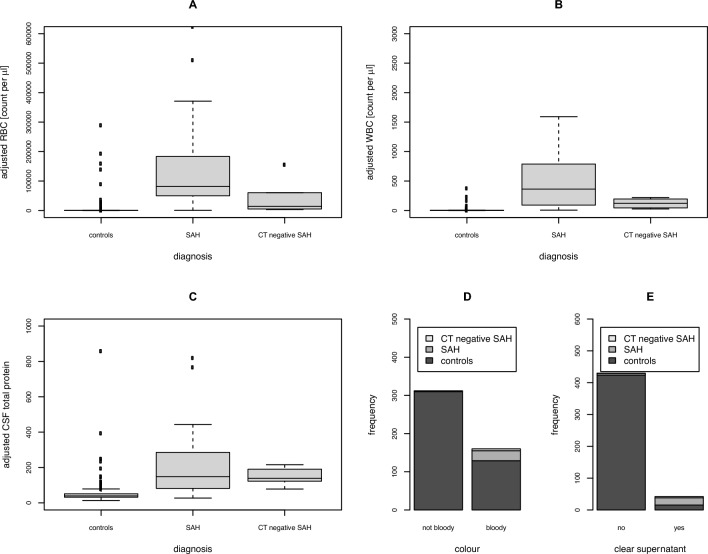
Various CSF parameters discriminating between SAH and traumatic LP. RBC count, WBC count and CSF total protein concentration were adjusted for a fixed disease duration of one day in patients with SAH and CT negative SAH. SAH and CT negative SAH patients showed higher RBC_adjusted_, WBC_adjusted_ and CSF total protein_adjusted_ compared to controls. Bloody CSF and xanthochromic supernatant were more frequently observed in patients with SAH and CT negative SAH than in controls. *CSF* cerebrospinal fluid, *CT* computed tomography, *RBC* red blood cells, *SAH* subarachnoid haemorrhage, *WBC* white blood cells.

RBC_adjusted_, WBC_adjusted_ as well as CSF total protein_adjusted_ were significantly higher in SAH patients and CT negative SAH patients compared to controls. SAH patients compared to controls showed more frequently bloody CSF (97% vs. 29%) and xanthochromic CSF supernatant (84% vs. 3%) (Fig. 2).

**Figure 3:**
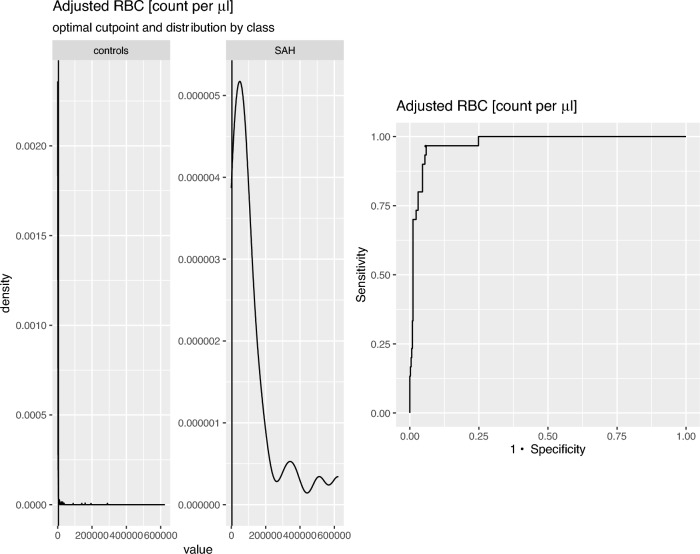
Determination of optimal cut point for CSF RBC count to identify SAH patients. Optimal cut point for CSF RBC count to identify SAH patients was 3667/µl. *RBC* red blood cells.

Multivariable logistic regression analyses identified bloody CSF colour [odds ratio (OR) 32.6, p = 0.013], xanthochromic CSF supernatant [OR 15.5, p < 0.001] and WBC_adjusted_ [OR 4.5 (per increase of 100/μl), p < 0.001] as predictors, while age, sex and CSF total protein_adjusted_ were not statistically significant predictors (Table 2). The high correlation between RBC_adjusted_ and WBC_adjusted_ is responsible for the lacking significance of RBC_adjusted_ as a predictor of SAH (Table S4). In univariate analysis, RBC_adjusted_ was a statistically significant predictor of SAH [OR 1.4 (per increase of 10,000/μl), p < 0.001] (Table S5). An optimal cut-point of RBC_adjusted_ count to identify SAH (both SAH and CT negative SAH) was obtained at 3667/µl (Fig. 3). Twenty-six of 27 (96.3%) SAH patients, and all CT negative SAH patients had a high RBC_adjusted_ (> 3667/µl), while 413 (94.1%) of 439 controls had low RBC_adjusted_ (≤ 3667/µl). The negative predictive value (NPV) of low RBC count was 99.8% in controls, while the positive predictive value (PPV) of high RBC was 54.4% considering all SAH patients (Table S6). Overall, patients who showed the combination of high RBC and CSF xanthochromia had SAH in 78.8% of cases, while all patients who showed low RBC and clear CSF supernatant were allocated to the control group (Table S6).

**Table 2:** Regression analysis including various CSF parameters.

	Estimate	Std. Error	Wald test		Likelihood Ratio test	
			z value	Pr ( >|z|)	Chi2	Pr (> Chi2)
Constant	−6.9160	2.0382	−3.393	0.0007***		
Sex (female)	−0.1642	0.7138	−0.230	0.8180	0.0530	0.8180
Age (years)	0.0004	0.0222	0.017	0.9864	0.0003	0.9864
RBC_adjusted_ (per 10,000 μl)	0.0680	0.0879	0.774	0.4389	0.6206	0.4308
WBC_adjusted_ (per 100 μl)	1.5011	0.4331	3.466	0.0005***	21.705	< 0.0001***
CSF total protein_adjusted_ (mg/dl)	−0.0059	0.0035	−1.669	0.0952	3.0928	0.0786
Colour = bloody	3.4839	1.9483	1.788	0.0737	6.2191	0.0126*
Supernatant = xanthochromia	2.7400	0.7959	3.443	0.0006***	12.990	0.0003***

McFadden R2: 0.7252.

Accuracy: 0.9724.

Sensitivity: 0.6875.

Specificity: 0.9932.

*CSF* cerebrospinal fluid, *RBC* red blood cell, *WBC* white blood cell.

*Indicates a p value < 0.05, ** < 0.01 and *** < 0.001.

Using RBC_adjusted_ (i.e. RBC counts adjusted for disease duration) instead of RBC_measured_ resulted in an improved classification of SAH patients versus controls of 11 percentage points (Table S7). The comparison of our RBC_adjusted_ cut-point with a previously suggested cut-point (for RBC_measured_ of 2000/μl) showed an increase in this classification by 15 percentage points (Table S8).

### Literature search

A total of 575 articles were screened. Of those, 29 focused on the diagnostic value of different CSF parameters in SAH for its differentiation from traumatic LP^14–42^. Two studies were excluded due to non-reporting appropriate results^20,38^. For a comprehensive overview, refer to Table S9. The majority of studies addressed RBC counts and xanthochromia with variable results^14–19,21–37,40–42^. Two recent studies not accounting for disease duration suggested a RBC cut-point of approximately 2,000 RBC/µl for detection of SAH and reported that low RBC in combination with clear CSF supernatant reliably excluded SAH^14,15^. There are also studies that assessed the decrease in RBC count between different CSF collection tubes^15,29,31^ with one study reporting that an approximately 60% decrease from first to final tube could be the optimal threshold to discriminate traumatic LP from SAH^29^. A few studies focused on erythrocyte degradation products such as CSF ferritin^39,40,43^ or bilirubin^30,32–37,40^. The latter has been recommended in clinical use in the U.K.^44^.

## Discussion

Bloody CSF obtained by LP of patients with suspected SAH but normal CT scan constitutes a diagnostic dilemma, as there is no single CSF parameter that allows a reliable discrimination between true SAH and traumatic LP. One of the main challenges in conducting studies and interpreting CSF results is that CSF biomarkers show different temporal dynamics^7^. Here, for the first time, we adjusted routine CSF findings for the individual disease duration at the time of LP and used the estimated values in a multivariable approach to identify predictors of SAH and traumatic tap, respectively. All patients with low adjusted CSF RBC counts and clear CSF supernatant were identified as traumatic LP and none as true SAH.^16^. This means that SAH can be excluded with very high certainty in patients fulfilling this CSF criterion.

To date, several studies have assessed the diagnostic value of different CSF parameters to identify SAH patients (Table S9)^14–43,45^. SAH occurs when blood is extravasated, in the majority of patients due to the aneurysm rupture^1^, and degradation of intrathecal blood leads to aseptic inflammation^46^. WBC are hypothesized to cross the arterial walls and to infiltrate the subarachnoid blood clot, secreting cytokines and initiating different processes^47,48^. Finally, blood and to a certain extent intrathecal inflammation lead to decreased CSF flow^49,50^. These main pathophysiological processes in SAH are mirrored in the CSF. Accordingly, we observed higher levels of RBC, WBC count and CSF total protein in SAH patients as compared to the control group confirming previous studies^14–16,26,28,51^.

Although different temporal dynamics of certain CSF biomarkers, e.g., of bilirubin or ferritin^7^, have been acknowledged as relevant factor in determining their diagnostic utility, none of the above-mentioned studies (Table S9) did this for routine CSF parameters such as RBC, WBC or CSF total protein. A recent study using several longitudinally collected CSF samples of SAH patients showed that RBC count is highest shortly after bleeding and gradually decreases over weeks. Similarly, WBC count and CSF total protein tend to normalize with advancing disease course^11^. We hypothesized that timing of LP impacts on the magnitude of the change of different CSF parameters. In the present study, CSF was taken a median of 9 days after disease onset in patients with SAH (mostly due to hydrocephalus) and after 6 days in patients with CT negative SAH. This is in contrast to patients with traumatic LP, where blood contamination of CSF occurs at the time of LP. In patients with SAH and CT negative SAH, RBC count at the time of the bleeding was estimated from the RBC count determined at the time of LP, using the previously published decay law^11^ and the respective time from symptom onset, i.e. adjusting for the in vivo cell degradation over time. Indeed, the measured and adjusted variables showed considerable differences (Table S2). This is clinically relevant because a significant proportion of patients with thunderclap headache—at risk of having suffered an intrathecal hemorrhage—present themselves with delay, sometimes several days^52^. Not considering disease duration would result in an underestimation of CSF changes due to the bleeding.

We identified an adjusted RBC cut point of approximately 3600/μl which was higher than previously suggested cut points^14,15^. Mark et al. observed that SAH patients (only 33% with LP performed within the first 12 h) had RBC counts above 2000/μl and/or xanthochromia^15^. Perry et al. included 15 CT negative SAH patients, which had LP up to 5 days after symptom onset and identified a RBC cut-off of 2450/μl^14^. However, both studies did not consider the different disease duration and, thus, used non-adjusted, potentially false-low RBC counts. In our study, when we used RBC_adjusted_ or RBC_measured_ as predictor of SAH versus controls, an improved classification was achieved by the RBC_adjusted_ approach.

In clinical practice, our approach would allow first to adjust the RBC count for the patient’s individual disease duration (i.e. time since symptom onset) and then to assess whether it is above or below the cut-off (i.e. to assess whether intrathecal bleeding is likely or not), instead of uncritically applying non-adjusted RBC counts, i.e. independent of disease duration, to a fixed cut-off. Despite of the obvious advantages of our approach, prospective studies are required to validate these results and to show superiority.

There are some limitations to our study. First, this was a retrospective study with all inherent limitations. Secondly, the decay rates used to adjust CSF parameters for individual disease duration were estimated in longitudinally collected CSF obtained by ventricular drainage^11^. However, we assume that cell degradation, which occurred in a non-linear manner, should be independent of the exact location within the CSF space and the site of the sample obtainment. Thirdly, we included CSF samples processed within 24 h after LP. Longer intervals to laboratory processing might result in decreased RBC and WBC count^9^. This means that shorter processing time would have led to higher RBC cut-off values. As the main conclusion of our study is that patients with RBC count below the cut-off are most likely traumatic LP, higher RBC cut-off would not impair the negative predictive values. Furthermore, we were not able to consider other CSF parameters such as bilirubin, or the “three-tube test” in our multivariable analysis, as we did not routinely perform or document these measurements. Also, some of the patients had an intervention (i.e. either coiling or clipping) before CSF withdrawal. It cannot be excluded that this had an influence on CSF parameters. We have to state that we adjusted CSF parameters in our analyses for a fixed disease duration of one day in SAH patients; an earlier time point would not be valid, as we also considered CSF supernatant, and colour change (into xanthochromia) needs at least 12 h as bilirubin has to be formed in vivo. Considering the disease duration within the SAH group of median 7.5 days (3. quartile: 16 days), it might be that at a different (earlier) time point of LP, a higher percentage of SAH patients would have shown CSF xanthochromia. However, it is not possible to adjust the variable “CSF xanthochromia” for disease duration, as in case of a negative status, a positive status before cannot be extrapolated. This means that the diagnostic value of CSF supernatant might have been underestimated. Another limitation of our study is that we could only include 5 patients with CT-negative SAH, therefore, the results on CT-negative SAH patients (e.g., the cut-off of RBC_adjusted_) need to be replicated by further studies.

Altogether, we present a tool, which has the potential to be widely used in clinical practice, to identify patients with traumatic LP by RBC count and inspection of CSF supernatant. Even though assessment of CSF supernatant is rater-dependent and anecdotal reports of SAH in patients with very low RBC counts^53^ cannot be excluded, the herein applied criterion (of an adjusted RBC count of less than 3667/μl combined with clear CSF supernatant) identified only patients with traumatic LP and none of the SAH patients. It has to be clearly stated that in case of high RBC counts and/ or xanthochromia, the differentiation between SAH and traumatic LP is still not reliable. Altogether, we think that the results of our study contribute to further understanding. Correct interpretation of CSF findings is of utmost importance, as SAH—if misdiagnosed—is a severe neurological disease with significant morbidity and mortality.

### Supplementary Information

Supplementary Tables.

## Data Availability

All data generated or analyzed during this study are included in this published article and its supplementary information files.
