# Supplementary material for: Subarachnoid haemorrhage or traumatic lumbar puncture. Differentiation by cerebrospinal fluid parameters in a multivariable approach
Source: Sci Rep. 2023 Dec 15;13:22310. doi: 10.1038/s41598-023-49693-y (PMC10724187; doi:10.1038/s41598-023-49693-y)
Supplement: Supplementary file 1 — Supplementary Tables. [file 41598_2023_49693_MOESM1_ESM.pdf]

Table S1: Diagnoses of non-SAH patients

	**Diagnosis**	**Frequency (n, %)**
Symptomatic controls[1]	Secondary headache (e.g., due to respiratory infection, hypertension)	71 (16.2)
	Tension headache	65 (14.8)
	Neck/ lower back pain	51 (11.6)
	Peripheral nerve compression syndrome	48 (10.9)
	Migraine	46 (10.5)
	Sensory disturbance	37 (8.4)
	Headache, not classified	27 (6.2)
	Somatoform disorder	22 (5.0)
	Dizziness/ vertigo	12 (2.7)
	Idiopathic facial nerve palsy	12 (2.7)
	Provoked seizure	5 (1.1)
	Transient limb weakness	4 (0.9)
	Visual disturbance	4 (0.9)
	Transient amnesia, disorientation or impaired consciousness	3 (0.7)
	Disturbed sleep rhythm	2 (0.5)
	Cluster headache	1 (0.2)
	Hemicrania	1 (0.2)
	Tremor	1 (0.2)
Non-neurological disease	Acute stress reaction	8 (1.8)
	Chronic fatigue syndrome	4 (0.9)
	Leukaemia[2]	4 (0.9)
	Lymphoma[2]	4 (0.9)
	Addiction	3 (0.7)
	Depression	2 (0.5)
	Attention deficit disorder	1 (0.2)
	Rheumatoid arthritis	1 (0.2)

Legend:

[1] Symptomatic controls were defined according to Teunissen et al. [8].

[2] Cerebral neoplastic manifestation excluded (cerebrospinal fluid collection in most cases before prophylactic intrathecal chemotherapy installation)

Abbreviations: n, number; SAH, subarachnoid haemorrhage

Table S2: Main routine CSF parameters before and after adjustment

	SAH (n=27)		CT negative SAH (n=5)		Control (n=439)
	**Measured value**	**Adjusted value**	**Measured value**	**Adjusted value**	**Measured value**
RBC count (/μl)	10000 (204-94375)	81496 (4517-2081730)	5120 (1012-68375)	13820 (3958-137160)	2 (1-4328)
WBC count (/μl)	28 (4-359)	363 (18-6550)	43 (22-71)	121 (29-214)	2 (0-10)
CSF total protein (mg/dl)	66 (26-618)	148 (47-1333)	130 (61-182)	139 (87-211)	40 (23-79)

Legend:

Data are shown as median (5-95th percentile) unless otherwise specified. RBC count, WBC count and CSF total protein concentration were adjusted for a fixed disease duration of one day in patients with SAH and CT negative SAH.

Abbreviations: CSF, cerebrospinal fluid; CT, computed tomography; RBC, red blood cell; SAH, subarachnoid haemorrhage; WBC, white blood cell

Table S3: Characterisation of CT negative SAH patients

**Diagnosis**[1]	**Thunderclap headache**	**Neck stiffness**	**Disease duration** (days)	**CSF colour**	**CSF supernatant**	**3-tube-test**	**RBC count (μl)**	**CSF cytology**	**Cerebral MRI**	**DSA**
CT negative SAH	Yes	Yes	1	bloody	xanth.	NA	5120	siderophages	NA	no aneurysm
CT negative SAH	Yes	Yes	8	bloody	xanth.	NA	21875	siderophages	negative[2]	ICA aneurysm[3] (surgery)
CT negative SAH	Yes	No	9	xanth.	xanth.	NA	1460	siderophages, hemosiderin	positive	ICA aneurysm[3] (coiling)
CT negative SAH	Yes	Yes	0	bloody	clear	Positive	75000	erythrophages	negative[2]	no aneurysm
CT negative SAH	Yes	No	6	bloody	xanth.	Positive	900	siderophages, hematoidin	negative[2]	no aneurysm

Legend:

[1] Diagnosis made by the treating physicians at that time.

[2] Brain imaging performed without susceptibility weighted imaging.

[3] intradural

Abbreviations: CSF, cerebrospinal fluid; CT, computed tomography; DSA, digital subtraction angiography; NA, not available; RBC, red blood cell; SAH, subarachnoid haemorrhage; xanth., xanthochromia

Table S4: Regression analysis including various CSF parameters considering the correlation between WBC and RBC counts

	Estimate	Std. Error	**Wald test**		
			**z value**	**Pr (>|z|)**	
Constant	-6.3957	1.9814	-3.228	0.0012	**
Sex (female)	-0.1642	0.7138	-0.230	0.8180	
Age (years)	0.0004	0.0222	0.017	0.9864	
$RBC_{adjusted}$ (per 10,000 μl)	0.2431	0.0912	2.667	0.0077	**
$WBC_{adjusted}$ (per 100 μl) #	1.5011	0.0043	3.466	0.0005	***
CSF total $protein_{adjusted}$ (mg/dl)	-0.0059	0.0035	-1.669	0.0952	
Colour = bloody	2.7400	0.7959	3.443	0.0006	***
Supernatant = xanthochromia	3.4839	1.9483	1.788	0.0737	

McFadden R2: 0.7252

Accuracy: 0.9724

Sensitivity: 0.6875

Specificity: 0.9932

Legend:

** indicates a p value <0.01 and *** <0.001

The linear effect of $RBC_{adjusted}$ on $WBC_{adjusted}$ is partialled out, i.e. the residuals of the regression of $WBC_{adjusted}$ on $RBC_{adjusted}$ are used.

Abbreviations: CSF, cerebrospinal fluid; RBC, red blood cell; WBC, white blood cell

Table S5: Univariate regression analyses of routine CSF parameters to discriminate SAH from controls

			Wald test	Likelihood Ratio test	
	Estimate	Std. Error	P value	Chi2	P value
Constant	-3.43405	0.26721	<0.001		
$RBC_{adjusted}$ (per 10,000 µl)	0.33900	0.05851	<0.001	85.316	< 0.001
McFadden R2	0.3648				

			Wald test	Likelihood Ratio test	
	Estimate	Std. Error	P value	Chi2	P value
Constant	-3.96045	0.33947	<0.001		
$WBC_{adjusted}$ (per 100 µl)	2.63500	0.49500	<0.001	132.700	< 0.001
McFadden R2	0.5674				

			Wald test	Likelihood Ratio test	
	Estimate	Std. Error	P value	Chi2	P value
Constant	-3.89217	0.32148	<0.001		
CSF total $protein_{adjusted}$ (mg/dl)	0.01574	0.00295	<0.001	61.212	< 0.001
McFadden R2	0.2617				

			Wald test	Likelihood Ratio test	
	Estimate		P value	Chi2	P value
Constant	-4.44030	0.44980	<0.001		
Supernatant (=xanthochromia)	5.02810	0.55320	<0.001	124.670	< 0.001
McFadden R2	0.5331				

			Wald test	Likelihood Ratio test	
	Estimate	Std. Error	P value	Chi2	P value
Constant	-5.73700	1.00200	<0.001		
Colour (=bloody)	4.31100	1.02100	<0.001	63.086	< 0.001
McFadden R2	0.2697				

			Wald test	Likelihood Ratio test	
	Estimate	Std. Error	P value	Chi2	P value
Constant	-2.84490	0.26560	<0.001		
Sex (=female)	0.47960	0.36730	0.192	1.707	0.191
McFadden R2	0.0073				

			Wald test	Likelihood Ratio test	
	Estimate	Std. Error	P value	Chi2	P value
Constant	-5.47261	0.70463	<0.001		
Age (year)	0.05569	0.01189	<0.001	23.915	< 0.001
McFadden R2	0.1023				

Legend:

Abbreviations: CSF, cerebrospinal fluid; RBC, red blood cell; WBC, white blood cell

Table S6: Diagnostic sensitivity, specificity, positive and negative predictive values to discriminate SAH from traumatic LP

(A)

CSF RBC count	SAH	CT neg. SAH	Controls	Total
**High**	26	5	26	57
**Low**	1	0	413	414
	27	5	439	471

Sensitivity	Sensitivity	Specificity
96.3	100	94.1
**PPV**	**PPV**	**NPV**
45.6	8.8	99.8

(B)

CSF supernatant	SAH	CT neg. SAH	Controls	Total
**Xanthochromia**	23	4	15	42
**Clear**	4	1	424	429
	27	5	439	471

Sensitivity	Sensitivity	Specificity
85.2	80.0	96.6
**PPV**	**PPV**	**NPV**
54.8	9.5	98.8

(C)

CSF RBC & Supernatant	SAH	CT neg. SAH	Controls	Total
**High and xanth.**	22	4	7	33
**High and clear**	4	1	19	24
**Low and xanth.**	1	0	8	9
**Low and clear**	0	0	405	405
	27	5	439	471

Sensitivity*	Sensitivity*	Specificity*
81.5	80.0	92.3
**PPV***	**PPV***	**NPV***
66.7	12.1	100

Legend:

A RBC count of 3667/μl was used to stratify into a high and low RBC category.

* Sensitivity, specificity, PPV and NPV are shown for patients with high CSF RBC & xanthochromic supernatant versus patients with low CSF RBC & clear supernatant.

Abbreviations

CT, computed tomography; neg., negative; RBC, red blood cell; SAH, subarachnoid haemorrhage; xanth., xanthochromic

Table S7: Comparison of $RBC_{adjusted}$ and $RBC_{measured}$ to identify patients with SAH and controls

(A)

**CSF $RBC_{adjusted}$**	**SAH**	**CT neg. SAH**	**Controls**	**Total**
**High**	26 (96%)	5 (100%)	26	57
**Low**	1	0	413 (94%)	414
	27	5	439	471

Correctly classified: 444/ 471 (94%)

(B)

**CSF $RBC_{measured}$**	**SAH**	**CT neg. SAH**	**Controls**	**Total**
**High**	24 (89%)	5 (100%)	77	106
**Low**	3	0	362 (82%)	365
	27	5	439	471

Correctly classified: 391/ 471 (83%)

Legend:

Optimal cut-points for $RBC_{adjusted}$ (3667/μl) and $RBC_{measured}$ (2880/μl) were determined by logistic regression to discriminate both SAH & CT negative SAH from controls, using the sum of specificity and sensitivity as optimization criterion.

Table S8: Comparison of different RBC cut-points to identify patients with SAH and controls

(A)

**CSF RBC$_{adjusted}$**	**SAH**	**CT neg. SAH**	**Controls**	**Total**
**High (≥3667)**	26<br>(96%)	5<br>(100%)	26	57
**Low (<3667)**	1	0	413<br>(94%)	414
	27	5	439	471

Correctly classified: 444/ 471 (94%)

(B)

**CSF RBC$_{measured}$**	**SAH**	**CT neg. SAH**	**Controls**	**Total**
**High (≥2000)**	24<br>(89%)	5<br>(100%)	97	126
**Low (<2000)**	3	0	342<br>(78%)	345
	27	5	439	471

Correctly classified: 371/ 471 (79%)

Legend:

Cut-point for RBC$_{adjusted}$ (3667/μl) was compared to previously published cut-point of RBC$_{measured}$ (2000/μl) to discriminate SAH from controls.

Table S9: Reports on various CSF parameters to discriminate spontaneous SAH from traumatic lumbar puncture

Reference	Disease groups	Number of patients	Age (years)	Sex (females)	Inclusion criteria	CSF collection site	CSF parameters	Cut-off	Sensitivity/ Specificity (%)
Perry et al. (2015) [14]	SAH	15	45 (±16)[#]	369 (58)	suspected SAH and LP;<br>conf. by CT/ X/ RBC in FT and A+	L	RBC,<br>xanthochromia[a,b]	RBC <2000/µl in FT	*RBC & xanthochromia*:<br>100 / 91
	Non-SAH	626							
Mark et al. (2015) [15]	SAH	64	52 (45-65)*	44 (69)	LP;<br>conf. by A	L	RBC,<br>xanthochromia[b]	RBC >2000/µl	*RBC:*<br>97 / n.sp.<br><br>*RBC & xanthochromia:*<br>100 / n.sp.
	Non-SAH	626	n.sp.						
Rankin et al. (2022) [16]	SAH	9	58	90 (61)	X analysis requested,<br>conf. by medical records	L	RBC,<br>xanthochromia[a]	RBC >1000/µl, xanthochromia according to UK NEQAS	*RBC & xanthochromia*<br>100 / 100<br><br>*Xanthochromia*:<br>100 / 94
	Non-SAH	1723	n.sp.	n.sp.					
Arora et al. (2010) [17]	SAH	19	n.sp.	n.sp.	medical charts review for SAH + LP;<br>conf. by CT/ CTA/ MRA	L	Xanthochromia[b]		47 / n.sp.
Hann et al. (2015) [18]	SAH	6	38 (±14)[#]	230 (56)	CT;<br>conf. by CTA/ MRI/ A	L	Xanthochromia[a,b]	See publication	*By visual inspection:*<br>50 / 99<br><br>*By visual inspection & spectrophotometry:*<br>100/ 80
	Non-SAH	403							
Perry et al. (2006) [19]	SAH	2	42 (±16)[#]	120 (55)	suspected SAH and LP;<br>conf. by CT/ RBC >5/µl in FT and A+/ X and A+	L	Xanthochromia[a,b]	Xanthochromia according to UK NEQAS	*By visual inspection:*<br>50 / 97<br><br>*By spectrophotometry:*<br>100 / 83
	Non-SAH	218							
Ahmed et al. (2014) [21]	SAH	2	n.sp.	n.sp.	X analysis requested;<br>conf. by CTA/ A/ MRA	L	Xanthochromia[a]	See publication	PPV 9 / NPV 100
	Non-SAH	21							

Dupont et al. (2008) [22]	SAH	13	48 (±16)[#]	99 (65)	suspected SAH, CT neg.; conf. by A	L	Xanthochromia[a]	See publication	93 / 95
	Non-SAH	139							
MacDonald et al. (1988) [23]	SAH	68	n.sp.	n.sp.	suspected SAH; conf. by A	L	RBC, Xanthochromia[b]	No RBC cut-off specified	*RBC:* 46 / n.sp<br>*Xanthochromia: 4/ n.sp*<br>*RBC + xanthochromia*: 37 / n.sp.
Wood et al. (2005) [24]	SAH	2	n.sp.	n.sp.	suspected SAH, CT neg.; conf. by A	L	Xanthochromia[a], XI	See publication	*Xanthochromia:* 100 / 75<br>*Xanthochromic index:* 100 / 71
	Non-SAH	251							
Rana et al. (2013) [25]	SAH	6	n.sp.	n.sp.	X pos.; conf. by CTA	L	Xanthochromia[a]	See publication	PPV 40
	Non-SAH	9							
Gangloff et al. (2015) [26]	SAH	5	49 (±6)[#]	3 (60)	X analysis requested, CT neg.; conf. by angiography/ X+/ RBC ≥5/µl in FT	L	Xanthochromia[a,b]	See publication	*By visual inspection:* 80 / 99<br>*By spectrophotometry:* 100 / 92-98 [c]
	Non-SAH	701	41 (±14)[#]	365 (52)					
Wallace et al. (2013) [27]	SAH	3	n.sp.	n.sp.	CT neg., CSF suggestive of SAH; conf. by A	L	Xanthochromia[b]		75 / 98
	Non-SAH	54							
Migdal et al. (2015) [28]	SAH	2	40 (30-55)*	200 (66)	suspected SAH, CT neg.; X/ RBC in FT & A+	L [d]	Xanthochromia	n.sp.	PPV 1
	Non-SAH	300							

Czuczman et al. (2013) [29]	SAH	26	50 (±13)[#]	21 (81)	suspected SAH, RBC ≥ 5/µl in FT; conf. by CTA/ MRA	L	RBC (in FT), RBC change (%, from initial to FT)	RBC (in FT) <100/µl<br>RBC change <63%	*RBC in FT*: AUC: 0.85 specificity 100%<br>*RBC change*: AUC: 0.84
	Non-SAH	196	43 (±15)[#]	112 (57)					
Tsementzis et al. (1985) [30]	SAH	99	49 (20-67)**	49 (50)	n.sp.	n.sp.	XI [a]	n.sp.	82 / n.a.
Heasley et al. (2005) [31]	SAH	8	n.sp.	n.sp.	suspected SAH, CT neg; conf. by A	L	RBC change (%, from initial to FT)	RBC change >25%	75 / 79
	Non-SAH	14							
Gunawardena et al. (2004) [32]	SAH	9	n. sp.	n. sp.	suspected SAH, CT neg.; conf. by MRI/ A	L	Bilirubin[a]	n.sp.	PPV 50[e]
	Non-SAH	454							
McCarron et al. (2015) [33]	SAH	10	49 (±20)[#]	8 (80)	elevated B; conf. by CT/ MRI/ A	L	Bilirubin[a]	n.sp.	PPV 23
	Non-SAH	46	45 (±21)[#]	16 (53)					
Bakr et al. (2017) [34]	SAH	4	41 (16-90)**	n.sp.	suspected SAH, CT neg.; conf. by CTA / A	L	Bilirubin[a]	n.sp	PPV 15
	Non-SAH	792							
Horstman et al. (2012) [35]	SAH	13	51 (27-73)**	14 (47)	suspected SAH, CT neg., elevated B; conf. by CT/ MRI/ A	L	Bilirubin[a]	n.sp.	43 / n.sp.
	Non-SAH	17							
Martin et al. (2015) [36]	SAH	5	45 (±18)[#]	94 (37)	suspected SAH, CT neg.; conf. by A/ medical records	L	Bilirubin, OX[a]	n.sp.	*Bilirubin & OX*: PPV 33
	Non-SAH	250							

Falconer et al. (2015) [37]	SAH	16	n. sp.	n. sp.	elevated B; conf. by CT/ medical records	L [d]	Bilirubin, OX[a],	n.sp.	*Bilirubin:* n. sp. / 44 [f] *Bilirubin & oxyhaemoglobin:* n. sp. / 60 [f]
	Non-SAH	28							
Watson et al. (2008) [39]	SAH	16 [q]	n.sp.	n.sp.	suspected SAH, CT neg., conf. by A/ medical records	L [d]	Ferritin	6.4 µg/l 12 µg/l	*Ferritin (6.4):* 100 / 48 *Ferritin (12):* 81 / 91
	Non-SAH	236							
O'Connell et al. (2003) [40]	SAH	10	n.sp.	n.sp.	CT neg.; conf. by A	L	Ferritin, Bilirubin[a]	n.sp.	*Ferritin:* 100 / 78 *Bilirubin:* 80 / 100
	Non-SAH	14							
Lang et al. (1990) [41]	SAH	6	n.sp.	n.sp.	n. sp.; conf. by CT/ MRI	L	D-Dimer, Xanthochromia[b]	n.sp.	*D-Dimer:* 100 / 100 *Xanthochromia:* 100 / 65
	Non-SAH	34							
Juliá-Sanchis et al. (2007) [42]	SAH	35	n.sp.	n.sp.	bloody CSF; conf. by CT	L	D-Dimer	0.5 mg/l	*D-Dimer:* 100 / 70
	Non-SAH	27							

Legend:

Data are shown as * median (interquartile range), ** median (range), or # mean ± standard deviation

a. Assessed by spectrophotometry.
b. Assessed by visual inspection.
c. Spectrophotometric analyses were performed using 2 methods: 1. UKNEQAS (specificity 98.1%) 2. Duiser iterative approach (specificity 91.9%).
d. CSF collection by LP was not clearly stated in the publication, but we assumed it due to the study design.
e. Only a proportion of patients underwent catheter angiography.
f. Diagnostic specificity given for patients with a CSF total protein concentrations <1 g/l.

Abbreviations: A, Angiography; B, Bilirubin; conf., confirmed; CSF, cerebrospinal fluid; CTA, computed tomography angiography; CT, computed tomography; FT, final tube; L, lumbar; MRI, magnetic resonance imaging; n.a.; not applicable; neg., negative; no., number; NPV, negative predictive value; n. sp.; not specified; OX, oxyhaemoglobin; pos., positive; PPV, positive predictive value; RBC, red blood cell; SAH, subarachnoid haemorrhage; X, xanthochromia; XI, xanthochromic index
